# The characteristics of androgen receptor splice variant 7 in the treatment of hormonal sensitive prostate cancer: a systematic review and meta-analysis

**DOI:** 10.1186/s12935-020-01229-4

**Published:** 2020-05-06

**Authors:** Zhize Wang, Haixiang Shen, Zhen Liang, Yeqing Mao, Chaojun Wang, Liping Xie

**Affiliations:** Department of Urology, The First Affiliated Hospital, College of Medicine, Zhejiang University, Hangzhou, 310000 China

**Keywords:** Androgen receptor splicing variant 7, First-line hormonal therapy, Prostatectomy, Prostate cancer, Predictor, Survival

## Abstract

**Background:**

Accumulating evidence suggests androgen receptor splice variant 7 (AR-V7) may be associated with the prognosis of castration-resistant prostate cancer (CRPC) received novel hormonal therapy while its characteristic and prognosis value in hormonal sensitive prostate cancer is unclear.

**Methods:**

We aimed to evaluate the prognostic role of AR-V7 by progression free survival (PFS) and overall survival (OS) in hormonal sensitive prostate cancer (HSPC), and the AR-V7-positive-proportion difference in HSPC and CRPC. A search of PubMed, Embase, and the Web of Science was performed using the keywords prostate cancer, prostate tumor, prostate neoplasm, prostate carcinoma; AR-V7, AR3, androgen receptor splicing variant-7, or androgen receptor-3. Seventeen trials published due December 2019 were enrolled.

**Results:**

AR-V7-positive proportion in CRPC was significantly larger than newly diagnosed prostate cancer (PCa) (odds ratio [OR] 7.06, 95% confidence interval [CI] 2.52–19.83, P < 0.001). Subgroup analyses indicated significantly higher AR-V7-positive proportion in CRPC derived from RNA in situ hybridization (OR 65.23, 95% CI 1.34–3171.43, P = 0.04), exosome RNA (OR 3.88, 95% CI 0.98–15.39, P = 0.05) and tissue RNA (OR 10.89, 95% CI 4.13–28.73, P < 0.001). AR-V7-positive patients had a significantly shorter PFS than those who were AR-V7-negative treated with first-line hormonal therapy (hazard ratio [HR] 3.63, 95% CI 1.85–7.10, P < 0.001) and prostatectomy (HR 2.49, 95% CI 1.33–4.64, P = 0.004). OS (HR 5.59, 95% CI 2.89–10.80, P < 0.001) were better in AR-V7-negative than AR-V7-positive HSPC patients treated with first-line hormonal therapy. The limitations of our meta-analysis were differences in study sample size and design, AR-V7 detection assay, and disease characteristics.

**Conclusion:**

AR-V7-positive proportion was significantly higher in CRPC than that in newly diagnosed PCa. AR-V7 positive HSPC patients portend worse prognosis of first-line hormonal therapy and prostatectomy. Additional studies are warranted to confirm these findings.

## Background

Prostate cancer (PCa) is the most common cancer in Western countries among male and the fifth leading cause of cancer death worldwide [1]. Most men eventually progress to castration-resistant prostate cancer (CRPC) which leads to poor prognosis [2]. Androgen receptor (AR) splice variants are identified as significant roles in the mechanisms of castration resistance [3, 4].

AR-V7 was firstly reported as an abnormally spliced mRNA isoform of the androgen receptor, which lacks the C-terminal ligand-binding domain but retains the transcriptional active N-terminal domain, and was constitutively active in driving the expression of androgen-responsive genes regardless of androgen level [5–7]. Accumulating evidences indicate that AR-V7 mRNA derived from circulating tumor cells (CTCs) may be a prognostic marker of novel hormonal therapy (NHT) resistance including Abiraterone and Enzalutamide [8–10], while could predict sensitivity to taxane chemotherapies such as docetaxel and cabazitaxel [9, 11]. After adjusting for physician propensity, the use of AR-V7 CTC test to inform treatment choice can improve patient outcomes relative to decisions based solely on standard-of-care measures [12]. Our previous meta-analysis concluded an association between AR-V7 positivity and poorer prostate specific antigen (PSA) response and PFS prognosis in CRPC patients treated with NHT, but not in chemotherapy, indicating new therapy decision strategy for CRPC patients [13].

Though growing evidences suggest the prognostic value of AR-V7 in CRPC, it remains unclear whether AR-V7 may also serve as a prognostic biomarker in hormonal sensitive prostate cancer (HSPC). Several studies reported the predictive role of AR-V7 in the first-line hormonal therapy outcomes in HSPC [14, 15]. Due to the diversity of patients’ cohort, sample characteristic, detection method, positivity and outcomes definition, the prognostic value and clinical utility of AR-V7 in HSPC are still under consideration. Moreover, various AR-V7 detection assays were reported, which were different in techniques, tissue type, and sampling criteria, may result in different interpretation of outcomes, limiting the validity and facticity usage of AR-V7 as a clinical prognosis biomarker of PCa.

This meta-analysis reviewed 17 clinical trials to integrated different measurements and compared the expression of AR-V7 in newly diagnosed PCa and CRPC, evaluated the treatment effectiveness of first-line hormonal therapy, and estimated the prognostic value of AR-V7 in HSPC. In order to understand the differences between various AR-V7 detection methods, subgroup analyses were further performed. The prognostic value was assessed by the impact on progression free survival (PFS) and overall survival (OS) of patients with different AR-V7 status. These are preliminary clinical evidences of the prognostic role of AR-V7 in HSPC, and further studies in larger cohorts are warranted.

## Methods

### Literature search

This meta-analysis was conducted out in accord with the Preferred Reporting Items for Systematic Reviews and Meta-Analyses (PRISMA) statement [16]. Search was completed before December 2019; published studies were retrieved from PubMed, the Web of Science, and Embase. The search terms included prostate cancer or prostate tumor or prostate neoplasm or prostate carcinoma; AR-V7 or AR3 or androgen receptor splicing variant 7 or androgen receptor 3. The references of the selected articles were also searched to identify additional eligible trials. Each study was assessed for inclusion by two or three independent reviewers. Discrepancies in the articles that were selected by the reviewers were resolved by discussion.

### Selection criteria

The titles and/or abstracts of the retrieved studies were screened, and the full text of those that satisfied the selection criteria were reviewed. Eligible studies met the following criteria. 1. The study reported on prostate cancer and AR-V7. 2. The results included AR expression and the proportion that consisted of AR-V7 (the AR-V7-positive proportion) in both newly diagnosed PCa and CRPC patients. Other reported results included PFS or OS after radical prostatectomy or first-line androgen deprivation therapy (ADT). 3. The results were obtained in a clinical trial, including prospective or retrospective cohort studies or comparative series. Studies were excluded if they 1. reported the AR-V7-positive proportion in, or only enrolled, newly diagnosed PCa or CRPC patients; 2. did not report PFS or OS; or 3. were animal or in vitro studies. 4. Studies in languages other than English were excluded unless a translator was available. 5. Case reports; letters; comments; editorials; and review papers were excluded. When more than one report of the same trial was available, the most recent information, with longer follow-up and a larger patient population were included in the analysis.

### Data collection and study quality

1. The patient characteristics extracted from each included trial included age, tumor stage, Gleason score, baseline PSA and alkaline phosphatase, and median time from diagnosis to sampling. 2. The description of study design included the country in which it was conducted, the treatment received, the primary end point, and the hypothesis tested. 3. The numbers of patients enrolled, assigned to treatment with radical prostatectomy or first-line ADT, and followed up, and the median follow-up time were extracted. 4. The AR-V7-positive proportion in CRPC and newly diagnosed PCa patient specimens and the AR-V7 detection assay were recorded. 5. Survival data for patients included the number of patients with PFS median PFS, and hazard ratios (HRs) with 95% confidence intervals (CIs), and P-values 6. OS: number of deaths in each study, median OS, HR with 95% confidence interval (CI), *P* value.

### Statistical methods

After data were abstracted, analysis was performed using Review Manager Software (RevMan v.5.3; The Nordic Cochrane Center, Copenhagen, Denmark). In all the included trials, efficacy data from all randomly assigned patients were analyzed on an intention-to-treat basis. The AR-V7-positive proportion in newly diagnosed PCa and in CRPC was evaluated. The primary end point of the meta-analysis was OS and the secondary end point was PFS.

The main analysis compared PFS and OS for first-line ADT in HSPC by different AR-V7 status. For both OS and PFS, the summary measure was HR (95% CI). A random effect model was applied. Statistical heterogeneity among studies was evaluated using the Chi square test and the I^2^ statistic. Odds ratio (OR) and HR estimates were weighted and pooled using the Mantel–Hansel random effect model. All statistical tests were two-sided, and statistical significance was defined as P < 0.05. No correction was made for multiple statistical testing.

## Results

### Characteristics of included studies

**Fig. 1:**
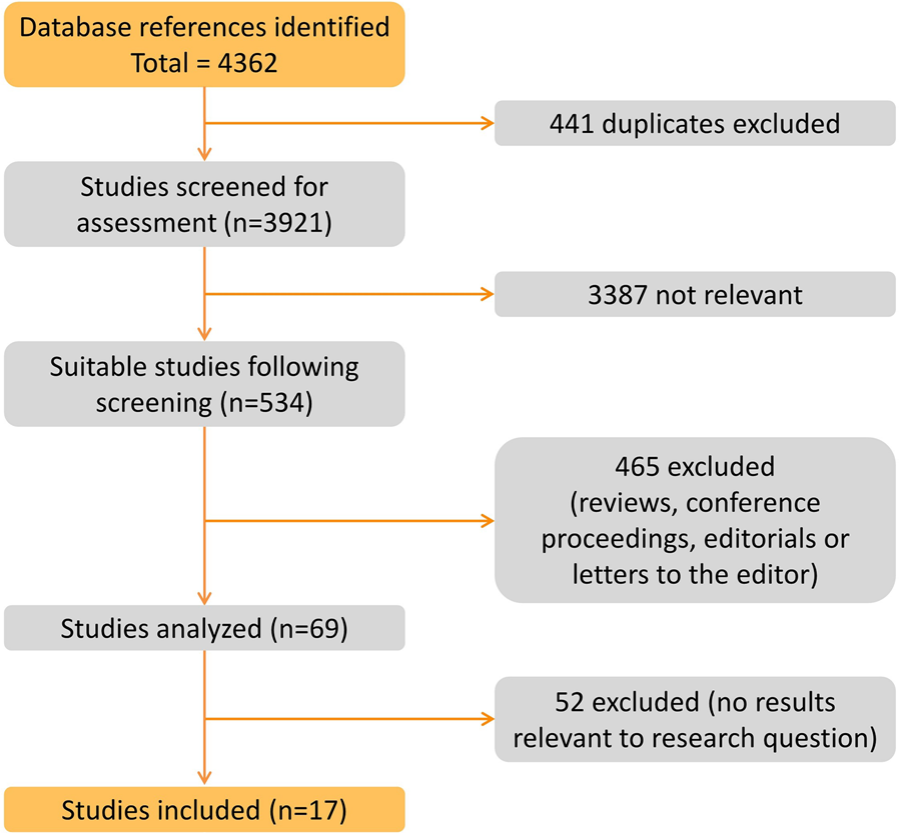
Study selection process

The study selection process is shown in Fig. 1. The search results were updated in December 2019, with the exclusion of 4345 of the 4362 full-length published papers. Briefly, 441 duplicated studies were excluded, 3387 irrelevant studies were excluded, 465 were conference abstracts, reviews, letters, and editorials that could not be quality assessed and thus were excluded, and 52 studies without relevant results were excluded. No studies from the reference lists added. The remaining 17 studies were included in the meta-analysis. Among which, 15 were included in the AR-V7-positive proportion analysis and 4 in the analysis of PFS and OS.

### Patient characteristics

Fifteen trials enrolling 1731 patients were included in the AR-V7-positive proportion meta-analysis, their characteristics are shown in Table 1. The target specimens and AR-V7 detection assays are shown in Additional file 1: Table S1 in detail. Four trials enrolling 518 patients were included in the PFS and OS meta-analysis. The characteristics of these studies and patients are shown in Table 2, and the definitions of PSA response, PFS, and OS, which differed among the trials, are shown in Additional file 1: Table S2.

**Table 1:** Characteristics of studies included in the AR-V7-positive proportion meta-analysis

Author	Year	Country		Newly diagnosed PCa				CRPC				
			AR-V7 detection assay	Patients (n)	Age (range)	Gleason score (range)	Median PSA (ng/ml) at sampling (range)	Patients (n)	Age (range)	Gleason score (range)	Median PSA (ng/ml) at sampling (range)	Follow-up time (month) Median (range)
Horenberg [19]	2011	Sweden	Tissue RNA	10	79 (60–85)	-	156 (21–10,000)	30	73 (54–88)	-	335 (4–5139)	-
Hu [6]	2009	USA	Tissue RNA	82	-	-	-	25	-	7 (5–9)	-	-
Qu [15]	2015	China	IHC	104	70 (43–84)	8	122.5 (3.0–6006.2)	46	65 (50–79)	8	98 (2.6–2350.0)	25 (2–132)
Welti [24]	2016	UK	IHC	33	-	-	-	35	67.5 (IQR64.2–75.3)	-	-	-
Zhang [32]	2011	USA	IHC	50	-	-	-	162	63 (42–93)	-	12.4 (1.7–4000)	-
Zhu [ 21]	2018	USA	RISH	9	-	-	-	28	64 (52-86)	-	59.6 (0.7-6746.8)	
		UK	RISH	0	-	-	-	16	72.3 (48.8-79.4)	-	177.0 (2.6-4098.0)	
Takeuchi [ 28]	2016	Japan	Whole blood RNA	20		-	-	23	-	-	-	-
Lee [30]	2017	Korea	Tissue RNA	13	-	7 (6-9)	12.76 (2.75-40.92)	3	65.3 (56-70)	8 (8-10)	8.78 (8.6-173.7)	-
Saylor [22]	2017	USA	RISH	30	-	7 (6–10)		12	-	-	-	-
Kallio [23]	2018	Finland	IHC/RNA-seq	24	-	7	6.3 (4.1-21.0)	30	-	-	-	-
Nimir [26]	2019	Australia	CTC RNA/ctRNA/Exosome RNA	12	-	-	-	32	-	-	-	-
Sharp [40]	2019	USA	IHC-									
			ICR/RMH	63	61.7 (SD=7.52)	>7 (67%)	51.7 (IQR 20.3-145.0)	160	68.5 (IQR 63.9-73.1)	-	230.5 (IQR 77.0-591.5)	-
			UW	128	60 (SD=8.21)	>7 (70%)	6.3 (IQR 3.3-67.0)	-	-	-	-	
Woo [29]	2018	Korea	Urinary Exosome RNA	22	70 (51-84)	>7 (14%)	1.01 (0.005-1667)	14	71.5 (60-82)	>7 (71.5%)	19.38 (0.006-646)	-
Park [31]	2019	Korea	Tissue RNA	19	65.9 (52-79)	>7 (58%)	-	19	-	>7(84%)	-	46(1-158)
Nakazawa [27]	2015	USA	CTC mRNA	2	53.5 (50–57)	9	339.5 (314–365)	12	67 (56-82)	>8 (91.7%)	159.5 (2.2-895)	11 (6–18)

*PCa* prostate cancer, *CRPC* castration resistance prostate cancer, *PSA* prostate specific antigen, *AR-V7* androgen receptor splicing variant 7, *CI* confidence interval

**Table 2:** Characteristics of studies and patients included in the PFS and OS meta-analysis

Study	Year	Country	Study design	AR-V7 detection assay	Patients characteristics						
					Treatment	Patients (n)	Age (range)	Gleason score (%)	Tumor stage at diagnosis (%)	Baseline PSA (ng/ml) median (range)	Follow-up time (month) Median (range)
Li H [14]	2018	China	Retrospective	Immunohistochemistry	First-line ADT	168	71 (53–96)	≤ 7 (35.7%) ≥ 8 (64.3%)	T1/T2 (9.5%) T3/T4 (67.9%)	118.78 (2.61–4003.40)	36 (IQR22–48)
Saylor [22]	2017	USA	Retrospective	RNA ISH	First-line ADT	22	–	–	–	–	–
Qu Y [15]	2015	China	Retrospective	IHC	First-line ADT	104	70 (43–84)	≤ 7 (44.2%) ≥ 8 (55.8%)	–	122.5 (3.0–6006.2)	25 (2–132)
Guo Z [5]	2009	USA	Retrospective	IHC	Radical prostatectomy	224	–	6–10	–	–	–

*IQR* inter quartile range, *SD* standard deviation, *AR-V7* androgen receptor splice variant 7, *CTC* circulating tumor cell, *PSA* prostate specific antigen, *ADT* androgen deprivation therapy, *ARS inhibitor* androgen receptor signal inhibitor, *LHRH* luteinizing hormone releasing hormone

### AR-V7-positive proportion in newly diagnosed PCa and CRPC

**Fig. 2:**
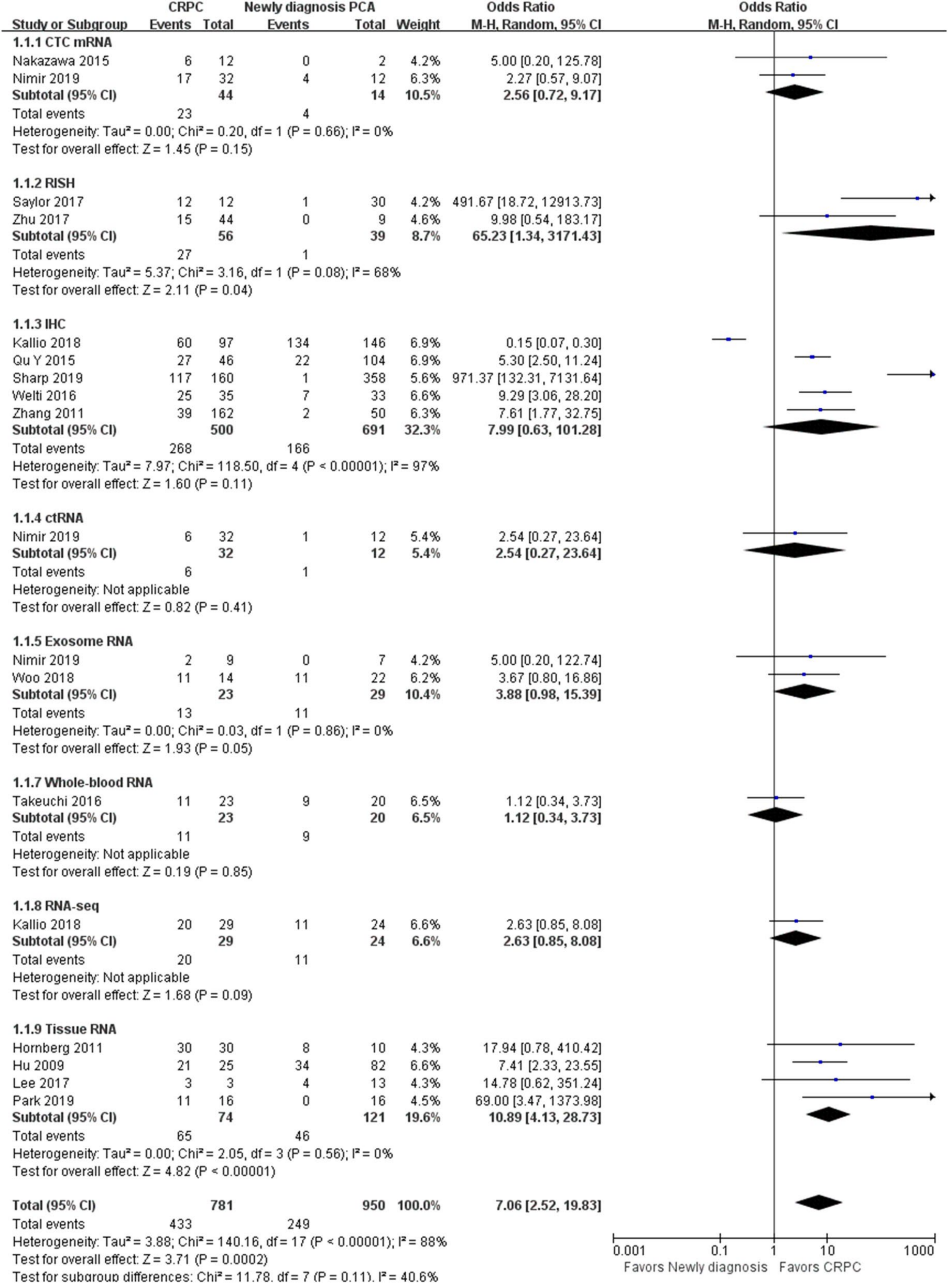
Forest plots of AR-V7-positive proportion in newly diagnosed PCa and CRPC from fifteen studies. AR-V7-positive proportion in newly diagnosed PCa and CRPC were calculated using random-effect models. Subgroup analyses were performed based on various AR-V7 detection assays. The bars indicate 95% CIs. *AR-V7* androgen receptor splicing variant 7, *CRPC* castration-resistant prostate cancer, *PCa* prostate cancer, *CI* confidence interval*, OR* odds ratio, *CTC* circulating tumor cell, *RISH* RNA in situ hybridization, *IHC* immunohistochemistry, *ctRNA* circulating tumor RNA

Overall, 433 of 781 CRPC and 249 of 950 newly diagnosed prostate cancer patients were AR-V7 positive. As shown in Fig. 2, the AR-V7-positive proportion was significantly higher in CRPC than in newly diagnosed PCa (OR 7.06, 95% CI 2.52–19.83, P < 0.001). As there was significant heterogeneity among the fifteen trials (P < 0.001, I^2^ = 88%), ORs and 95% CIs were calculated by a random-effects model. Subgroup analysis of different AR-V7 detection assays was further performed, and there was no significant subgroup differences (I^2^ = 40.6%, p = 0.11). The AR-V7-positive proportion was significantly higher in CRPC derived from RNA in situ hybridization (RISH) (OR 65.23, 95% CI 1.34–3171.43, P = 0.04), exosome RNA (OR 3.88, 95% CI 0.98–15.39, P = 0.05) and tissue RNA (OR 10.89, 95% CI 4.13–28.73, P < 0.001) while other detection assays showed no statistical difference.

### The progression free survival in HSPC of different AR-V7 status

**Fig. 3:**
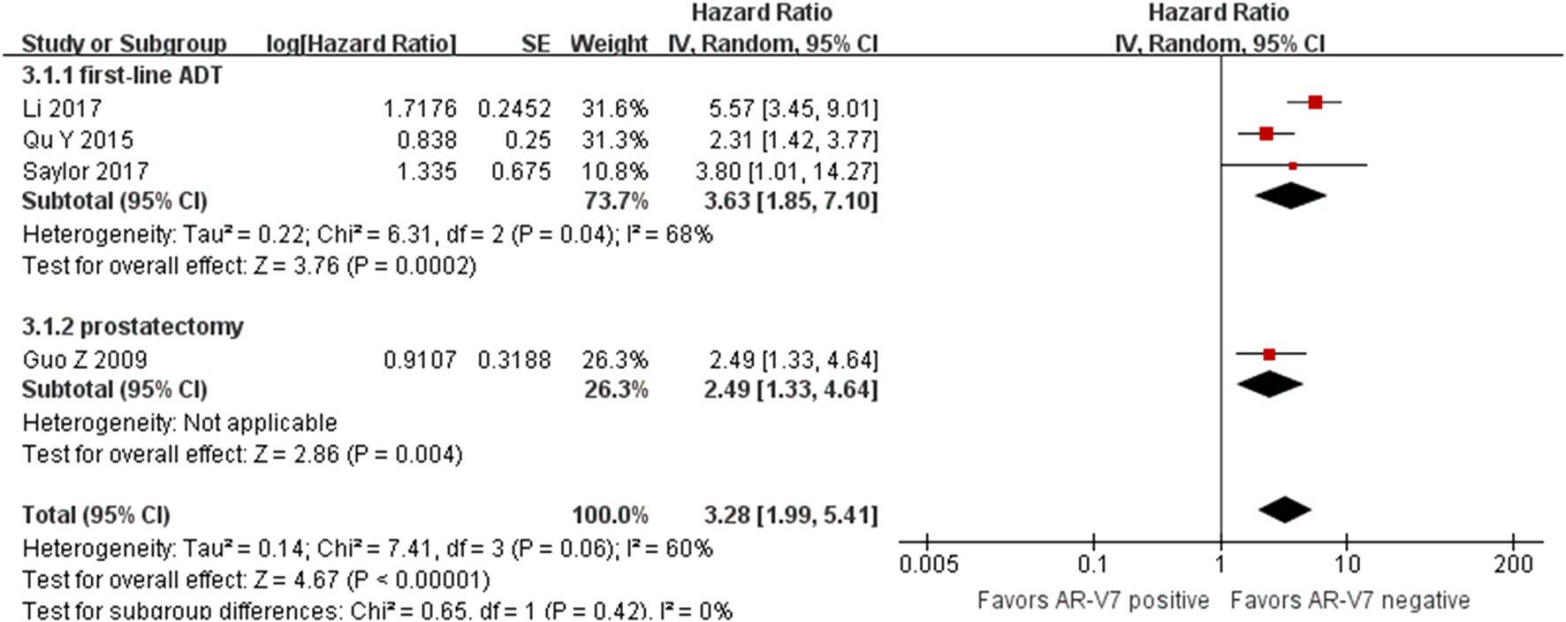
Forest plots of hazard ratios (HRs) for PFS of first-line and radical prostatectomy in HSPC patients respectively with different AR-V7 status. Pooled HRs were calculated using random effect. The bars indicate 95% CIs. *ADT* androgen deprivation therapy, *AR-V7* androgen receptor splicing variant 7, *CI* confidence interval, *PFS* progression free survival

In the entire study population (Fig. 3), the AR-V7-negative HSPC patients had a significant PFS benefit compared with the AR-V7-positive patients (HR 3.28, 95% CI 1.99–5.41, P < 0.001). There was significant study heterogeneity (I^2^ = 60%, P = 0.06), so the random effect model was applied to calculate the HR and 95% CI. The subgroup analysis found that the HR for the PFS of AR-V7-positive patients was 3.63 (95% CI 1.85–7.10, P < 0.001) in patients treated with first-line ADT, 2.49 (95% CI 1.33–4.64, P = 0.004) in patients received radical prostatectomy, and there was no significant subgroup differences (I^2^ = 0%, p = 0.42).

### The overall survival in HSPC of different AR-V7 status

**Fig. 4:**
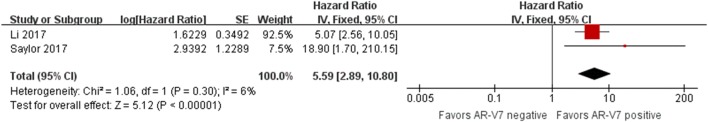
Forest plots of hazard ratios (HRs) for OS of first-line in HSPC patients with different AR-V7 status. Pooled HRs was calculated using fixed effect. The bars indicate 95% CIs. *ADT* androgen deprivation therapy, *AR-V7* androgen receptor splicing variant 7, *CI* confidence interval, *OS* overall survival

As shown in Fig. 4, the entire study population was treated with first-line ADT. The AR-V7-negative HSPC patients had a significant OS benefit compared with the AR-V7-positive patients (HR 5.59, 95% CI 2.89–10.80, P < 0.001). As there was no significant study heterogeneity (I^2^ = 6%, P = 0.30), a fixed effects model was used to calculate the HR and 95% CI.

## Discussion

First-line hormonal therapy has been the standard-of-care for metastatic and locally advanced prostate cancer, but most patients eventually progress to castration-resistant prostate cancer within 2–3 years, which is a lethal stage of the disease. [17, 18]. Therefore, it is necessary to identify a clinical marker which could predict the reaction to hormonal therapy and the risk of castration-resistant prostate cancer progression. We processed this meta-analysis to verify a hypothesis that AR-V7 expression elevated after CRPC progression, and had a poor prognosis in HSPC treated with first-line hormonal therapy or prostatectomy respectively. AR-V7 would thus be a clinical biomarker for the prognosis of hormonal therapy in hormonal sensitive prostate cancer. Various studies have found that AR-V7 is a novel ARs variant that can initiate and promote CRPC growth [5–7]. As AR-V7 is known to be associated with the pathogenesis of CRPC, the prognostic value of AR-V7 in HSPC needs to be elucidated.

We evaluated differences in the AR-V7-positive proportion in newly diagnosed prostate cancer and in CRPC. This systematic review and meta-analysis demonstrated that the AR-V7-positive proportion is frequently up-regulated in CRPC as compared to newly diagnosed PCa (OR 7.06 95% CI 2.52–19.83, P < 0.001), and may emerge as an adaptive response to therapies targeting the AR-signaling axis, which is consistent with previous reports [5, 6, 13]. A number of methods have been used to detect AR-V7 in PCa, making use of its unique exon composition and exon–exon junction, AR-V7 can be reliably detected by reverse transcription polymerase chain reaction (RT-PCR) [19]. Consequently, quantitative reverse transcription polymerase chain reaction (qRT-PCR) is preferred as the detection of AR-V7 in cell cultures and tissue specimens, especially in CTC, a non-invasive test for the analysis of AR-V7 expression developed by Antonarakis et al. [8]. Although qRT-PCR provides a highly sensitive and specific assay for the detection of AR-V7 in CTC, the presence of AR-V7 mRNA does not always correlate with AR-V7 protein expression and has lower sensitivity in formalin-fixed paraffin-embedded tissue [19, 20]. Other promising methods to analyze AR-V7 like RISH or RNA sequencing are presented [21–23]. Prior clinical studies using AR-V7 specific antibodies in immunohistochemistry (IHC) showed that the AR-V7 protein is commonly up-regulated in CRPC and rises as an adaptive response to therapies targeting the canonical AR signaling axis [5, 15, 19, 24], but the limited number of AR-V7 antibodies, the optimization and standardization of IHC interpretation method restricted its clinical use. Subgroup analyses was further performed according to various AR-V7 detection assays in our study, significant positive-proportion elevation was noted in RISH (OR 65.23 95% CI 1.34–3171.43, P = 0.04), RNA derived from exosome (OR 3.88 95% CI 0.98–15.39, P = 0.05) and tissue RNA (OR 10.89 95% CI 4.13–28.73, P < 0.001). Although the findings are consistent with the role of AR-V7 in castration resistance, more studies are warranted to validate this analysis and to assay AR-V7 expression in HSPC tissue.

PFS and OS were evaluated to compare the prognosis of first-line hormonal therapy and prostatectomy in HSPC respectively with different AR-V7 status. Four studies were included, and treatment was analyzed in subgroups. AR-V7-positive patients had an increased risk of worse OS compared with AR-V7-negative patients of first-line hormonal therapy; same as PFS was associated with AR-V7 status in HSPC treatment. Since Antonarakis et al. [8–10] and Scher et al. [25] reported that AR-V7 in circulating tumor cells is associated with NHT resistance and poor survival in CRPC but not significantly affect OS in patients treated with chemotherapy, there were rising researches about the AR-V7 detection and its prognostic value of hormonal therapy. AR-V7 has been a hot-spot prognostic role and potential therapeutic target of CRPC. Furthermore, the prognostic role of AR-V7 in first-line hormonal therapy in HSPC patients also raised concern. Our previous cohort study shows a dramatically worse outcomes of ADT in AR-V7 positive patients with markedly lower CRPC progression-free survival (HR 5.571, 95% CI: 3.445–9.007, P < 0.0001) and overall survival (HR 4.667, 95% CI: 2.382–9.142, P < 0.0001) [14]. Similar results were reported by Qu et al. [15] and Saylor et al. [22].

Our systematic review has limitations. The statistical power was limited by the small and distinct sample sizes of these studies, which ranged from 16 to 224 participants. Several factors might have contributed to seemingly contradictory results reported in these studies. Firstly, smaller studies certainly lead to less reliability because of the size effect and portend to publication bias. Funnel plots shown in Additional file 1: Figures S1–S3 clearly show an asymmetrical distribution of studies with low statistical power. Secondly, study designs differ greatly. Many studies samples were enrolled from a single center, which lead to an unclear selection bias. Thirdly, several different AR-V7 detection assays were used to determine AR-V7 positivity, including qRT-PCR of mRNA derived from CTC [26, 27], whole blood [28], exosome [26, 29], or representative tissue [6, 19, 30, 31], IHC [5, 14, 15, 23, 24, 32], RISH [21, 22] and RNA sequencing [23]. The advantages and disadvantages of different AR-V7 detection assays are discussed in an authoritative review [33]. The cut-off value is essential in the interpretation of morphologically AR-V7 detection such as RISH or IHC, while the continuous values lead to differences in detection rate between studies. Moreover, detection assays differ in sample type, tissue quality, and sampling criteria may lead to different results. Although studies showed some promise, a common limitation with AR-V7 antibody is that the detection methods may not be validated enough due to suboptimal detection sensitivity/specificity. While more studies are needed to precisely quantify the clinical validation of individual and integrated assays, it is feasible to measure AR-V7 and other AR aberrations using blood-based assays [34]. Details of the target specimens and AR-V7 detection assays used in the meta-analysis are listed in Additional file 1: Table S1. Last but not the least, disease characteristics including stage and metastasis location, the definition of PFS and OS vary among the studies, and might be responsible for the study heterogeneity that we reported. It must be acknowledged that the study of AR-V7 is not particularly extensive, especially about the AR-V7 prognosis value in HSPC, which would most likely lead to controversial conclusions.

We made several important efforts to handle with the limitations. First, a systematic, comprehensive, and reproducible search strategy was applied for the relevant studies in multiple online databases to minimize publication bias. Secondly, the eligibility criteria were clear and critical to limit bias from the varieties in AR-V7 detection methods, determination of cutoff points and definition of prognosis. Selection bias was acknowledged, but we believe that it was minimized by our specific restrictions of the qualification of studies in each step of the meta-analyses. Thirdly, further subgroup analyses of results concluded from different AR-V7 detection assays were performed. Fourthly, study design details, disease stage, AR-V7 detection method, types of therapy, baseline PSA, and follow-up period are tabulated and available for further analysis and reference (Tables 1 and 2).

The individual optimal therapy strategy of prostate cancer remains highly concern. Early combination of docetaxel to androgen deprivation therapy in hormone-sensitive metastatic prostate cancer indicated benefit in several randomized clinical trials [35–38]. Clinical validated markers are eagerly needed to address suitable patients because of the additional chemotherapy toxicity. Several markers have been found, but none suggest the selection of prostate cancer treatment [39]. This systematic review offers a comprehensive overview of the prognosis value of AR-V7 in HSPC treatment, that AR-V7 might be a potential therapy target and prognostic biomarker in HSPC patients. Therefore, it is important to develop prospective trials to further assess the clinical utility of AR-V7 in HSPC with the potential to improve the appropriateness of treatment.

## Conclusions

In conclusion, our meta-analysis clearly showed the AR-V7-positive proportion was significantly higher in CRPC than that in newly diagnosed prostate cancer. AR-V7 positive HSPC patients portend worse prognosis of first-line hormonal therapy and prostatectomy as shown by PFS and OS. AR-V7 might be a predictive biomarker in HSPC, indicates more aggressive and AR-V7 targeted therapy strategies to AR-V7-positive patient. Expanded, cross-institutional studies designed to further validate AR-V7 as a treatment selection marker is warranted and future studies aimed to improve AR-V7 measurements to assess the clinical utility of AR-V7 in HSPC are expected.

## Supplementary information

**Additional file 1: Figure S1.** The funnel plot for meta-analysis of the AR-V7 positive proportion in newly diagnosis prostate cancer and CRPC. **Figure S2.** The funnel plot for meta-analysis of the progression free survival of first-line hormonal therapy in HSPC of different AR-V7 status. **Figure S3.** The funnel plot for meta-analysis of the overall survival of first-line hormonal therapy in HSPC of different AR-V7 status. **Table S1.** Target specimens and AR-V7 detection assay of studies in the positive proportion meta-analysis in newly diagnosed PCa and CRPC. **Table S2.** Definition of PSA response, PFS and OS in the studies included in the meta-analysis of prognosis for hormonal therapy and chemotherapy in different AR-V7 states.

## Data Availability

The datasets used in this study are available from the corresponding author upon reasonable request.

## Abbreviations

AR-V7: Androgen receptor splice variant 7
PFS: Progression free survival
OS: Overall survival
HSPC: Hormonal sensitive prostate cancer
CRPC: Castration-resistant prostate cancer
PCa: Prostate cancer
OR: Odds ratio
CI: Confidence interval
HR: Hazard ratio
AR: Androgen receptor
CTC: Circulating tumor cell
NHT: Novel hormonal therapy
PSA: Prostate specific antigen
ADT: Androgen deprivation therapy
qRT-PCR: Quantitative reverse transcription polymerase chain reaction
RISH: RNA in situ hybridization
IHC: Immunohistochemistry
