# Supplementary material for: The characteristics of androgen receptor splice variant 7 in the treatment of hormonal sensitive prostate cancer: a systematic review and meta-analysis
Source: Cancer Cell Int. 2020 May 6;20:149. doi: 10.1186/s12935-020-01229-4 (PMC7201592; doi:10.1186/s12935-020-01229-4)
Supplement: Supplementary file 1 — Additional file 1: Figure S1. The funnel plot for meta-analysis of the AR-V7 positive proportion in newly diagnosis prostate cancer and CRPC. Figure S2. The funnel plot for meta-analysis of the progression free survival of first-line hormonal therapy in HSPC of different AR-V7 status. Figure S3. The funnel plot for meta-analysis of the overall survival of first-line hormonal therapy in HSPC of different AR-V7 status. Table S1. Target specimens and AR-V7 detection assay of studies in the positive proportion meta-analysis in newly diagnosed PCa and CRPC. Table S2. Definition of PSA response, PFS and OS in the studies included in the meta-analysis of prognosis for hormonal therapy and chemotherapy in different AR-V7 states. [file 12935_2020_1229_MOESM1_ESM.docx]

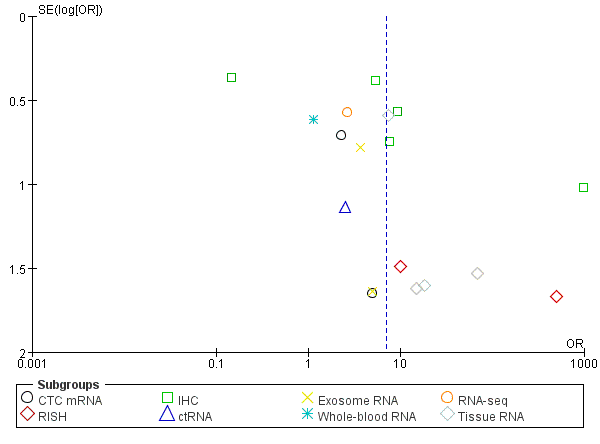


**Additional file 1: Figure S1 – the funnel plot for meta-analysis of the** **AR-V7 positive proportion in newly diagnosis prostate cancer and CRPC**


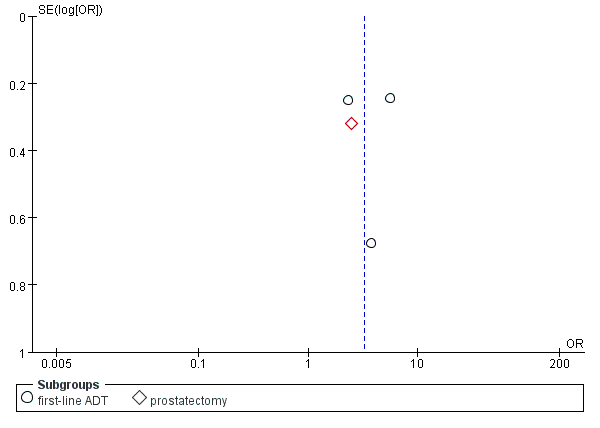


**Additional file 1: Figure S2 – the funnel plot for meta-analysis of the progression free survival of first-line hormonal therapy in HSPC of different AR-V7 status**


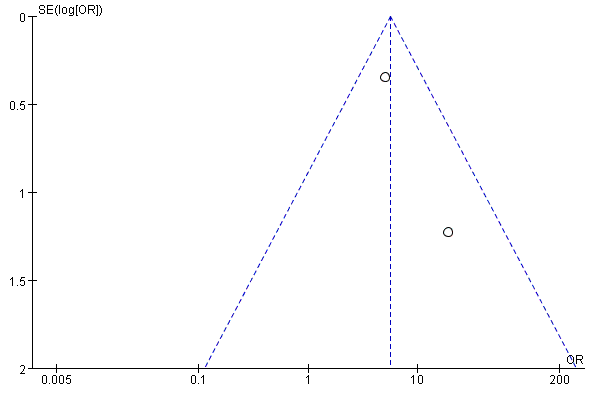


**Additional file 1: Figure S3 – the funnel plot for meta-analysis of the overall survival of first-line hormonal therapy in HSPC of different AR-V7 status**

**Additional file 1: Table S1 – Target specimens and AR-V7 detection assay of studies in the positive proportion meta-analysis in newly diagnosed PCa and CRPC.**

| Study | Target specimens | AR-V7 detection assay |
| --- | --- | --- |
| Hornberg ([1](#_ENREF_1)) | RNA extraction by Trizol protocol from representative prostate tissue | qRT-PCR  Housekeeping gene Ribosomal protein L30, normalized to the median value for the primary tumors samples |
| Hu ([2](#_ENREF_2)) | RNA extraction by Trizol protocol from RRP/TURP/ autopsy | qRT-PCR  Housekeeping gene SF3A3,  normalized to the median value for the RRP samples |
| Qu ([3](#_ENREF_3)) | FFPE PCa tissue | Immunohistochemistry.  Anti-AR-V7 antibody (A&G Pharmaceutical, Columbia, MD, USA). Cores with an immunostaining score of 0 or 1 were considered negative and score ≥ 2 were considered positive |
| Saylor([4](#_ENREF_4)) | FFPE tissues | RNA ISH  Target the unique cryptic exon CE3 Automated ISH assays for mRNA were performed using ViewRNA eZl Detection Kit (Affymetrix, Santa Clara, CA) |
| Welti ([5](#_ENREF_5)) | FFPE matched archival and CRPC tissue | Immunohistochemistry.  A rabbit monoclonal antibody (EP343) detected AR-V7, but not AR-FL or ARV567es. |
| Zhang ([6](#_ENREF_6)) | Human tissue microarrays from autopsy/RRP/TURP | Immunohistochemistry.  C-terminal AR antibody(Santa Cruz, C-19) N-terminal AR antibody(Santa Cruz,441; Biogenex, F39.4.1)  Variations in N-terminal and C-terminal AR expression: N+C↓(C-terminal AR score dropped more than 50% compared to N-terminal in nucleus) |
| Zhu, Y.([7](#_ENREF_7)) | FFPE tissues | RNA ISH  The BaseScope assays[18_TD$DIF] (Advanced Cell Diagnostics, Inc., Hayward, CA) for AR-FL/AR-V7 were developed to achieve junction-specific detection of the AR transcripts. |
| Takeuchi, T.([8](#_ENREF_8)) | RNA extraction by Trizol protocol from whole blood | qRT-PCR  Thirty-fve cycles of polymerase chain reactions (PCRs) (primary PCR) in 50 µL were performed using a Premix Taq DNA Polymerase (Takara Bio, Inc.) for the amplifcation of AR-V7 |
| Lee([9](#_ENREF_9)) | FFPE tissues | Immunohistochemistry  Rabbit monoclonal anti-AR-V7 antibody (ab198394, Abcam, Cambridge, UK) |
| Saylor PJ.([10](#_ENREF_10)) | FFPE tissues | RNA ISH  The AR-V7 RNA ISH probe was designed to target the unique cryptic exon CE3 as identified in the NCBI nucleotide database, using multiple tiling probes. |
| Kallio([11](#_ENREF_11)) | RNA extraction by Trizol protocol from representative prostate tissue | Whole genome and whole transcriptome/ HiSeq |
| Nimir([12](#_ENREF_12)) | CTCs isolated from blood samples | cDNA of either CTC, ctRNA or exosome samples was used for detection of AR-V7 transcripts by ddPCR. the reference gene GAPDH. |
| Sharp([13](#_ENREF_13)) | FFPE tissues | A recombinant rabbit monoclonal antibody Clone RM7) was developed against CE3 of AR-V7. AR-V7 positive defined as H score >10. |
| Woo([14](#_ENREF_14)) | urine derived extracellular vesicles (EVs) mRNA | The miRNeasy kit (Qiagen, Hilden, Germany) was used to extract total RNA from EVs isolated from 4 mL urine by UC and Exo-Hexa. AR-V7 mRNA levels were simultaneously quantified using the QX200 ddPCR system (Bio-Rad, Hercules, CA,USA).3.8 copies per mL as the cut-off value |
| Park([15](#_ENREF_15)) | FFPE tissues | Total RNA was extracted using the ReliaPrepTM FFPE Total RNA Miniprep System. A NanoString nCounter Digital Analyzer (NanoString Technologies, Seattle, WA, USA) was used to count the digital barcodes for the quantification of target mRNA in each sample based on the geometric mean of the three housekeeping genes (ACTB, ALAS1, HPRT1). FFPE RNA >250 Nanostring count defined as high AR-V7 mRNA expression |

FFPE=formalin fixed paraffin embedded tissue, RRP= radical prostatectomy, TURP= transurethral resection of prostate, RNA ISH = RNA *in situ* hybridization, qRT-PCR= Quantitative real-time polymerase chain reaction,

**Additional file 1: Table S2 –Definition of PSA response, PFS and OS in the studies included in the meta-analysis of prognosis for hormonal therapy and chemotherapy in different AR-V7 states**

| study | PSA response | Progression free survival | Overall survival |
| --- | --- | --- | --- |
| Li H([16](#_ENREF_16)) | PSA response (PSA level ≤ 4 ng/ml, maintained for ≥ 4 weeks) at 6 and 7 months of treatment | an increase in the PSA level by 25% or more above the nadir (and by ≥ 2 ng/ml), with confirmation four or more weeks later (PCWG3 criteria). | the time to death due to any cause |
| Saylor PJ.([10](#_ENREF_10)) |  | The date of the start of ADT to the date of biochemical disease progression. biochemical castration resistance (defined as a confirmed rise in PSA from the nadir despite testosterone <50 ng/dL) | the time to death due to any cause |
| Qu Y([3](#_ENREF_3)) |  | the time of diagnosis until CRPC confirmation | the interval between the TURP date (at castration-resistant stage) and the date of death or the last follow-up visit for censored patients. |
| Guo Z ([17](#_ENREF_17)) | - | a PSA elevation of >0.2 ng/mL after radical prostatectomy with successive increasing PSA values. | - |

IQR= inter quartile range; SD= standard deviation; ALP = alkaline phosphatase; AR-V7 = androgen receptor splice variant 7; CTC = circulating tumor cell; PSA = prostate-specific antigen; PCWG2=Prostate Cancer Working Group 2

1. Hornberg E, Ylitalo EB, Crnalic S, et al. Expression of androgen receptor splice variants in prostate cancer bone metastases is associated with castration-resistance and short survival. PloS one. 2011;6(4):e19059. Epub 2011/05/10.

2. Hu R, Dunn TA, Wei S, et al. Ligand-independent androgen receptor variants derived from splicing of cryptic exons signify hormone-refractory prostate cancer. Cancer research. 2009;69(1):16-22. Epub 2009/01/02.

3. Qu Y, Dai B, Ye D, et al. Constitutively active AR-V7 plays an essential role in the development and progression of castration-resistant prostate cancer. Scientific reports. 2015;5:7654. Epub 2015/01/08.

4. Saylor PJ, Lee RJ, Arora KS, et al. Branched chain RNA in situ hybridization for androgen receptor splice variant AR-V7 as a prognostic biomarker for metastatic castration-sensitive prostate cancer. Clinical cancer research : an official journal of the American Association for Cancer Research. 2016. Epub 2016/07/22.

5. Welti J, Rodrigues DN, Sharp A, et al. Analytical Validation and Clinical Qualification of a New Immunohistochemical Assay for Androgen Receptor Splice Variant-7 Protein Expression in Metastatic Castration-resistant Prostate Cancer. European urology. 2016. Epub 2016/04/28.

6. Zhang X, Morrissey C, Sun S, et al. Androgen receptor variants occur frequently in castration resistant prostate cancer metastases. PloS one. 2011;6(11):e27970. Epub 2011/11/25.

7. Zhu Y, Sharp A, Anderson CM, et al. Novel Junction-specific and Quantifiable In Situ Detection of AR-V7 and its Clinical Correlates in Metastatic Castration-resistant Prostate Cancer. European urology. 2018;73(5):727-35. Epub 2017/09/04.

8. Takeuchi T, Okuno Y, Hattori-Kato M, Zaitsu M, Mikami K. Detection of AR-V7 mRNA in whole blood may not predict the effectiveness of novel endocrine drugs for castration-resistant prostate cancer. Research and reports in urology. 2016;8:21-5. Epub 2016/02/13.

9. Lee CH, Ku JY, Ha JM, et al. Transcript Levels of Androgen Receptor Variant 7 and Ubiquitin-Conjugating Enzyme 2C in Hormone Sensitive Prostate Cancer and Castration-Resistant Prostate Cancer. The Prostate. 2017;77(1):60-71. Epub 2016/08/24.

10. Saylor PJ, Lee RJ, Arora KS, et al. Branched Chain RNA In Situ Hybridization for Androgen Receptor Splice Variant AR-V7 as a Prognostic Biomarker for Metastatic Castration-Sensitive Prostate Cancer. Clinical cancer research : an official journal of the American Association for Cancer Research. 2017;23(2):363-9. Epub 2016/07/22.

11. Kallio HML, Hieta R, Latonen L, et al. Constitutively active androgen receptor splice variants AR-V3, AR-V7 and AR-V9 are co-expressed in castration-resistant prostate cancer metastases. British journal of cancer. 2018. Epub 2018/07/11.

12. Nimir M, Ma Y, Jeffreys SA, et al. Detection of AR-V7 in Liquid Biopsies of Castrate Resistant Prostate Cancer Patients: A Comparison of AR-V7 Analysis in Circulating Tumor Cells, Circulating Tumor RNA and Exosomes. Cells. 2019;8(7). Epub 2019/07/11.

13. Sharp A, Coleman I, Yuan W, et al. Androgen receptor splice variant-7 expression emerges with castration resistance in prostate cancer. The Journal of clinical investigation. 2019;129(1):192-208. Epub 2018/10/20.

14. Woo HK, Park J, Ku JY, et al. Urine-based liquid biopsy: non-invasive and sensitive AR-V7 detection in urinary EVs from patients with prostate cancer. Lab Chip. 2018;19(1):87-97. Epub 2018/12/01.

15. Park HK, Lim SD, Kwon GY. mRNA expressions of androgen receptor and its variants in matched hormone-sensitive and castration-resistant prostate cancer. Scand J Urol. 2019:1-7. Epub 2019/12/07.

16. Li H, Wang Z, Xiao W, et al. Androgen-receptor splice variant-7-positive prostate cancer: a novel molecular subtype with markedly worse androgen-deprivation therapy outcomes in newly diagnosed patients. Mod Pathol. 2018;31(1):198-208. Epub 2017/10/28.

17. Guo Z, Yang X, Sun F, et al. A novel androgen receptor splice variant is up-regulated during prostate cancer progression and promotes androgen depletion-resistant growth. Cancer research. 2009;69(6):2305-13. Epub 2009/02/27.
